# Characterization of the clonal profile of methicillin resistant *Staphylococcus aureus* isolated from patients with early post-operative orthopedic implant based infections

**DOI:** 10.1186/s12941-019-0307-z

**Published:** 2019-02-13

**Authors:** Sonia Jain, Rukhsana Chowdhury, Mousumi Datta, Goutam Chowdhury, Asish Kumar Mukhopadhyay

**Affiliations:** 1 Infectious Diseases & Immunology Division, CSIR-Indian Institute of Chemical Biology, Kolkata, India; 2 Department of Community Medicine, Medical College & Hospital, Kolkata, India; 3 Division of Bacteriology, National Institute of Cholera and Enteric Diseases, Kolkata, India

**Keywords:** MRSA, Orthopedic implants, Post-operative infections, Epidemiology

## Abstract

**Background:**

To analyze the molecular epidemiology and to compare between the major methicillin resistant *Staphylococcus aureus* biotypes for association with patient characteristics who had an implant for closed fracture and developed early post-operative wound infections (POWI) in a tertiary care hospital of India.

**Methods:**

Pulsed-field gel electrophoresis (PFGE), antimicrobial resistance, accessory gene regulator (*agr*) and staphylococcal cassette chromosome *mec* (SCCmec) types, Paton–Valentine leukocidin (PVL) gene, toxin gene profiling, biofilm formation and patient demographics were correlated with MLST clonal complexes (CC).

**Findings:**

Overall eight different sequence types (STs) were detected with a predominance of ST239 (66%), ST22 (18%) and some minor types ST772, ST30 (4% each) ST1, ST642, ST6, ST107 (2% each). All ST239 isolates belong to CC239 and SCC*mec* III whereas ST22 isolates belong to CC22 and SCC*mec* IV. The isolates varied in the distribution of various toxin genes. With 63.63% biofilm formers ST239 were all multidrug resistant with frequent resistance to erythromycin, clindamycin, gentamicin, cefuroxime, amoxyclav and ciprofloxacin indicating doxycycline, amikacin, vancomycin and linezolid can be the drug of choice.

**Conclusion:**

This study shows that ST239 MRSA is still most prevalent strain with new emergence of ST642 and ST107 isolates in association with orthopedic implant based POWI. As compare to other ST types ST239 strain was associated with adverse treatment outcomes. This highlights the importance of improving nosocomial infection control measures in this unit.

## Introduction

*Staphylococcus aureus* is the leading cause of early post-operative wound infections (POWI) in patients undergoing implant based orthopedic procedures [1, 2]. In orthopedic devices, biofilm acts as a reservoir for bacteria, making eradication difficult which leads to prolong antibiotics use, implant rejection, removal of orthopedic hardware therefore increasing healthcare cost [3, 4]. Symptom associated *S. aureus* infections are caused by typical toxins [5] which are regulated by accessory gene regulator (*agr*) [6]. The first methicillin resistant *S. aureus* (MRSA) infections appeared in 1961 and have since become a major worldwide nosocomial pathogen. However, epidemiology of MRSA changed with the first report of community-associated MRSA (CA-MRSA) occurred in 1981 [7]. Till now eleven types of staphylococcal cassette chromosome SCC*mec* have been characterized on the basis of nucleotide variations of the *mec* gene complex, cassette chromosome recombinase (*ccr*) complex and junkyard regions [8]. With the emergence of multidrug resistant MRSA strains, demand for global surveillance and antimicrobial stewardship strategies increases [7, 9]. In order to distinguish strains within a heterogeneous species for local epidemiologic investigation purposes, multilocus-sequence typing (MLST) and pulsed-field gel electrophoresis (PFGE) is required which group MRSA strains into different clones [10, 11].

The prevalence of ST239-MRSA-III, ST22-MRSA-IV and ST772-MRSA-V in India has already been reported in global epidemiologic trials [12–14]. At present, an increasing number of reports from India and worldwide about the potential epidemiological shift indicate that CA-MRSA strains are gradually replacing HA-MRSA strains in hospitals [12, 13].

The purpose of this study was to investigate the type of MRSA clones causing early (< 3 month) post-operative orthopedic implant based infections through MLST, PFGE, SCC*mec*, *agr* and toxin gene typing schemes. In resource poor settings like in India, where there are major hurdles in acquiring relevant clinical data these results will help clinicians to decide the future treatment strategy and infection control policies for new and existing strain.

## Materials and methods

### Patients profile

After obtaining Ethical clearance and informed consent from the patients, 50 nonduplicate MRSA isolates were recovered from cases which were selected for a period of 2 years from Feb 2015 to Jan 2017 among the patients who were either admitted or came for follow-up in the Department of Orthopedics, Medical College and Hospital, Kolkata. Therefore, all those patients who had a history of closed wound fractures requiring implant based fixation and developing infection due to MRSA at surgical site within 3 months of surgery were included. The criteria for the diagnosis of POWI were according to the National Research Council of USA. The wound was examined on post operative days 3rd, 7th and 14th and subsequent follow-up of patients. Excluded were patients developing infection with pathological fracture managed with external fixators, diabetic or any other immune compromised state, isolates showing mixed infections or infections other than MRSA.

### Bacterial isolates and antimicrobial susceptibility testing

The 50 MRSA isolates from pus were stored in glycerol broth at − 80 °C. *S. aureus* was confirmed by standard laboratory methods [15]. All the confirmed *S. aureus* strains were further screened for methicillin resistance with the help of cefoxitin discs (30 μg/disc) (Oxoid) by using Kirby–Bauer disc diffusion method [15, 16]. DNA extraction was performed using DNA isolation kit (Qiagen 12888-100) with slight modifications. Then further to confirm the resistance, *mecA* PCR was done. Antimicrobial susceptibility patterns were performed by disc diffusion methods as per Clinical and Laboratory Standards Institute (CLSI) guidelines [16].

### Biofilm formation

By the modified quantitative microtitre plate method in vitro biofilm formation was determined as previously described [17]. *Staphylococcus epidermis* ATCC35984 and ATCC 12228 were used as positive and negative controls, respectively. Optical density (OD) of stained adherent biofilm was obtained by using micro ELISA autoreader (model 680, Biorad, UK) at wavelength 490 nm. Blank-corrected absorbance values were used for reporting biofilm production. The experiment was performed in triplicate and repeated three times.

### SCCmec, agr and toxin genes typing

The SCCmec types and *agr* four (I–IV) major specificity groups were determined by multiplex PCR using a previously published method [8, 18]. All the MRSA isolates were also tested for the presence of SE genes *sea, seb*, *sec, sed, see, seg, seh, sei, sej*, *sek*, *sel*, *sem*, *sen*, *seo*, *sep*, *seq*, *ser* and *seu* [19, 20] exfoliatin toxin genes *eta, etb* [21], toxin shock syndrome *tsst* gene [19] and the panton valentine leucocidin lukS/F-PV gene [5].

### MLST

MLST was performed for all MRSA isolates based on the sequencing of internal fragments of seven housekeeping genes in *S. aureus* [22]. Sequence types (STs) were assigned using the *S. aureus* MLST database (http://www.mlst.net). Clonal complexes (CC) were determined using the eBURST algorithm.

### PFGE

PFGE using *Sma*I (New England BioLabs) restriction enzyme was performed for all the MRSA isolates using previously described method [23]. Electrophoresis was performed in the PFGE CHEF-Mapper electrophoresis cell (Bio-Rad California, USA) by the contour clamped homogeneous electric field method by using a 50–400 kb size range under the following conditions: initial switch 6.76 s, final switch 35.38 s, at 6 V/cm at 14 °C for 19 h. *Xba I* digested DNA of *Salmonella enteric* serovar Braenderup (H9812) was used as control and size standards for the running parameters. The patterns of DNA fingerprint were analyzed using BioNumerics 4.0 software (Applied Maths, Sint-Martens-Latem, Belgium). The band patterns among different isolates were compared using Dice coefficients with a 1.5% band position tolerance. A dendrogram of PFGE results was created using the unweighted pair group method with arithmetic averages (UPGMA). The cluster cutoff was set at 80% similarity [24].

### Statistical analyses

The statistical analysis was carried out with SPSS software version 17 (SPSS Inc., Chicago, IL, USA). For continuous variables unpaired Student’s *t*-test and for categorical variables Pearson Chi square test was done. The results were considered statistically significant if *p* ≤ 0.05 (two-tailed).

## Results

### Patient populations

A total of 174 patients having orthopedic implant based early POWI were included in this study. The most common organism isolated was *S. aureus* 79 (45.4%) of which 50 (63.29%) were MRSA and rest of the infections were due to other pathogenic microorganisms.

Orthopedic implants and prosthesis were used for the arthroplasty and fracture fixation cases. In the arthroplasty group (n = 6/50; 12%) MRSA was isolated from infected implants in case of THK (n = 4) or TKR (n = 2). In the fracture fixation (n = 44/50; 88%) devices group the infected implants included plates/screws (n = 24), intramedullary nails (n = 9), hip screws (n = 4), other screws (n = 2) and tension band wiring (n = 5).

### MLST

In the 50 MRSA isolates examined, eight different known STs were detected. The most common STs found in the present study were ST239 (n = 33; 66%) and ST22 (n = 9; 18%), ST772 and ST30 were detected in two isolates each and three other STs were found (ST1, ST642, ST6, ST107), each corresponding to one isolate. STs were grouped in CC by using eBURST. The vast majority of STs belonged to CC239 (33/50; 66%). The next predominant was CC22 (9/50; 18%), CC30 and CC1 (3/50; 6%) and one isolate belongs to CC6 (1/50; 2%). The results indicated that the predominant ST type in MRSA strains examined in the present study was ST239 followed by ST22. In this study two known STs (ST642, ST107) were detected first time in MRSA isolates from India (Table 1).

**Table 1:** Molecular typing, virulence, resistance and source data for all MRSA isolates included in the study (N = 50)

Sequence type (no. of isolates)	CC or singleton	PFGE CLUSTERS (A–I) (no. of clusters)	SCC*mec* and *agr* typing (no. of isolates)		Biofilm formation	Number of isolates positive for:			Source (s) (no. of isolates)
			SCC*mec*	*agr*		*tsst*	*enterotoxins*	*pvl*	
ST239 (33)	239	D, E, F, H, I and G	III	I	BF (21)	0	*sea* (19), *sek* and *seq* (33)	0	Plates and screws (14) THR (4), TKR (2) Intramedullary nails (8), other screws and wires (4), hip screw (1)
ST22 (9)	22	A, B	IV	I	BF (5)	2	*egc* (9) *sea* (3)	9	Plates and screws (7) Wire (1) Hip screw (1)
ST30 (2)	30	C	IV	III	NBF (2)	0	*egc* (2)	2	Hip screw (1), intramedullary nail (1)
ST642 (1)	30	C	IV	III	NBF (1)	0	*sea*, *egc* (1)	1	Hip screw (1)
ST772 (2)	1	G	V	II	BF (1)	0	*sea* (2)*, egc* (2) *sec* (1)*, sel* (1)	2	Wire (1) Plate and screw (1)
ST1 (1)	1	–	V	III	NBF (1)	1	*sea, she, sek*, *seq* (1)	1	Plate and screw (1)
ST6 (1)	6	–	IV	I	BF (1)	0	*sea* (1)	0	Plate and screw (1)
ST 107 (1)	–	–	NT	NT	BF (1)	1	*egc* (1)	0	Screw (1)

*ST* sequence type, *CC* clonal complex, *S* singleton, *NT* non typeable

### Antibiogram of MRSA isolates

**Fig. 1:**
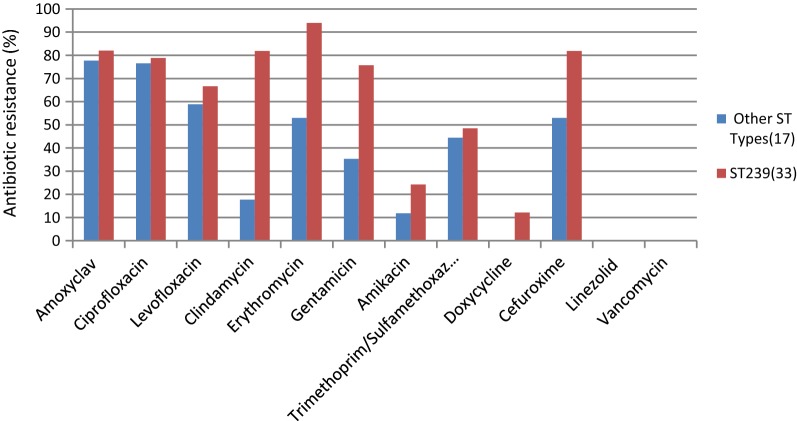
Comparative resistance pattern of ST239 against other ST types

The antimicrobial susceptibility pattern of the MRSA strains was examined. Overall the number of resistant isolates was highest for amoxyclav (84%), erythromycin (82%), ciprofloxacin (80%), levofloxacin (72%), cefuroxime (70%) followed by clindamycin and gentamicin (62%), trimethoprim–sulfamethoxazole (40%), amikacin (20%). Relatively fewer isolates were resistant to doxycycline 12%. All isolates were susceptible to the antibiotics vancomycin and linezolid. Though all the isolates of ST239 and ST22 were multidrug resistant (MDR), the spectrum of drug resistant for ST239 was wider (Fig. 1). ST772, a known CA-MRSA was multidrug resistant where as strains of ST1 and ST6 were relatively susceptible to various antibiotics.

### Biofilm formation

Biofilm formation was examined in all the isolates using microtitre plate method. Out of 50 MRSA isolates, 29 (58%) were found to be biofilm producers of which 17 (34%) were strong biofilm producers, 12 (24%) were moderate biofilm producers. ST239 isolates were high biofilm formers as compare to the other ST types (*p* = 0.028).

### Distribution of SCC*mec* and *agr* types and toxin genes

The distribution of the SCC*mec* types, agr and toxins combination among the 50 isolates is given in (Table 1). Majority of the isolates (n = 33; 66%) carried SCC*mec* III genes. These included isolates of ST239. SCC*mec* IV was detected in all the isolates (n = 13; 26%) of ST22, ST30, ST642 and ST6 and SCC*mec* V in ST772 and ST1 (n = 3; 6%).

Predominant agr types among the 50 isolates was agr I (n = 43; 86%) comprising of ST239, ST22 and ST6 isolates followed by *agr* III (n = 4; 8%) in the ST642, ST30, ST1 isolates and *agr* II (n = 2; 4%) in ST772. Only one isolate belonging to ST107 was non typeable for both *agr* and SCC*mec* types with the pair of primers used. In this study no isolates belonging to *agr* type IV was detected. A correlation between SCCmec and agr types could not be established as agr I included isolates with SCCmec III and IV. agr III included isolates with SCCmec IV and V while SCCmec V included isolates with agr II or III.

It was found that the most prevalent enterotoxin gene in the isolates was *sek* and *seq* gene (n = 34; 68%) followed by *sea* gene (n = 27; 54%), *egc* gene cluster (n = 15;30%). Exotoxins *pvl* (n = 16; 32%) and *tsst* (n = 4; 8%) were also detected. Of the two predominant ST types all the ST22 isolates carried *pvl*, *egc* gene cluster and only few of its isolates carried the *sea* and *tsst* gene. *pvl*, *egc* and *tsst* was not present in any of the 33 isolates belonging to ST239 but *sea, sek* and *seq* gene was variably present. The toxins *seb, sed, see, sej*, *sep, ser, seu*, *eta* and *etb* were not found in any of the isolates.

All the 50 MRSA strains contain multiple toxin genes except 1 strain belonging to CC6, which contains only *sea*. The most prevalent SE gene combination was sea-sek-seq, carried by 19 MRSA strains in CC239 and 1 strain in CC1 with additional presence of *seh*, *pvl* and *tsst* gene. The combination of *pvl*, *sea* and *egc* gene cluster was found in 3 strains belonging to CC22 and 2 strains of CC1of which 1 contained an additional SE genes (*sec* and *sel*). An isolate of ST772-MRSA-III belonging to CC1 showed the presence of maximum exotoxin genes (n = 9) *sea, sec, seg*, *sei*, *sem*, *sen*, *seo*, *sel*, PVL. The tsst gene was detected in very few isolates, co-existed with different kinds of SE genes of various CCs (Table 1). Therefore, the conserved prevalence of exotoxin genes in clinical MRSA strains shows the transfer of SE gene encoding mobile genetic elements (MGEs), which leads to increase pathogenicity of the isolates.

### PFGE analysis

Genetic typing by PFGE grouped all the 46 strains into nine clusters based on 80% similarity as a cutoff. Each cluster was designated by a letter from A–I. Strain N315 belonging to CC5 was kept as control. In each cluster, the number of strains varied. The largest PFGE pattern was identified in cluster D (n = 13/48; 27%) contained MRSA-19, 2, 20, 4, 34, 42, 43, 44, 14, 31, 33, 35, 36 of which MRSA 19, 2, 20, 4 and 42, 43 showed 100% similarity. Cluster D, E, F, H, I comprise of only CC239 strains with G containing 2 MRSA isolates (MRSA 27, 28) one belonging to CC239 and other to CC1 respectively. Therefore, the majority of the clusters belong to CC239 (Table 1). Cluster B has 4 MRSA isolates (MRSA 47, 21, 48, 40) of which MRSA 47, 21, 48 shows 100% similarity with all strains belonging to CC22 complex. In cluster C, 3 isolates were present (MRSA 10, 12, 37) of which two isolates of ST 30 shows 100% similarity and remaining 1 isolate of ST642 was 85% similar, all these strains belongs to CC30.

The PFGE types were generally associated with unique antibiotic resistance profiles as shown in (Table 2). In addition to unique common profiles individual strains in each category showed resistance to some additional antibiotics (data not shown). Six isolates belonging to PFGE type D shows additional resistance to antibiotic DO. Similarly, isolates belonging to G and I clusters were resistant to AMK. From the analysis most emergent multidrug resistant strain belongs to CC239 under which D type shows resistance to maximum number of antibiotics CIP, LVX, ERY, CLI, SXT, DOX, GEN, AMC, CXM. In contrast one isolate of B type belonging to CC22 was least antibiotic resistant CIP, ERY, AMC.

**Table 2:** PFGE types of MRSA isolates included in the study and their respective antibiotic resistance profiles

PFGE clusters (no. of isolates)	MRSA isolates	Predominant antibiotic resistance profiles
A (2)	MRSA 30, 5	CIP, LVX, ERY, CLI, CXM
B (4)	MRSA 21, 47, 48, 40	CIP, AMC
C (3)	MRSA 10, 12, 37	CIP, SXT, AMC, CXM
D (13)	MRSA 19, 2, 20, 4, 34, 42, 43, 44, 14, 31, 33, 35, 36	ERY, GEN, AMC, CXM
E (3)	MRSA 11, 41, 45	GEN, CXM
F (3)	MRSA 49, 7, 8	ERY, CLI, SXT, GEN, AMC, CXM
G (2)	MRSA 27, 28	CIP, LVX, SXT, AMK, GEN, AMC
H (3)	MRSA 17, 9, 3	CIP, SXT
I (2)	MRSA 23, 25	CIP, LVX, ERY, AMK, GEN, CXM

Zones of inhibition falling into the category of intermediate susceptibility to a particular antibiotic were considered resistant

Antimicrobial resistance patterns of MRSA from the laboratory. *CIP* ciprofloxacin, *LVX* levofloxacin, *GEN* gentamicin, *ERY* erythromycin, *SXT* trimethoprim–sulfamethoxazole, *AMK* amikacin, *DOX* doxycycline, *CLI* clindamycin, *AMC* amoxyclav, *CXM* cefuroxime

### Association of MRSA ST types with patient characteristics

In this study all the ST239 strains showed high multidrug-resistant phenotypes, high biofilm formation and increased severity of the infections as they lead to frequent cases of infected implant failure, re-implantation with lower cure rate (Table 3).

**Table 3:** Association of MRSA isolates with patient characteristics in early SSI of orthopedic implants

All types of implant infections (N = 50)	ST239 MRSA (*n* = 33)	ST22 MRSA and others (*n* = 17)	Comparison ST239 MRSA vs. ST22 and other ST types *p*-value*
Patient population: female	11 (36.37%)	5 (29.4%)	0.2
Mean age	40.21 years	37.35 years	0.5
Obesity (BMI > 30)	8 (24.24%)	4 (23.52%)	0.9
Mean time between implantation and onset of infection	14.21 days	11.29 days	0.16
Mean duration of antibiotics (antibiotic exposure)	28.51 days	19.58 days	0.000*
Elective surgery	8 (24.24%)	2 (11.76%)	0.2
Mean no. of surgical interventions (repeated debridement)	2.75	2.52	0.37
Removal of infected implant	26 (78.79%)	8 (47.05%)	0.02*
Re-implantation of a new implant	21 (63.64%)	6 (35.29%)	0.05*
Cure	23 (69.69%)	13 (76.47%)	0.00*
Mean length of hospital stay	34.9 days	27.41 days	0.02*

*Statistically significant *p*-values of ≤ 0.05 (two-tailed)

## Discussion

Surgical site infection due to MRSA is one of the main reasons for revision arthroplasty and fracture fixation procedures [1, 2, 25, 26]. This is the first observational study from India enclosing the molecular and clinical epidemiology, virulence properties and antibiogram pattern of MRSA strains isolated from these cases.

In this study the MRSA isolated belonged to six clonal complexes (CC239, CC22, CC1, CC30, CC6 and ST107) but the majority (84%) corresponded to the most prevalent ST239-MRSA-III and ST22-MRSA-IV clones. The oldest pandemic CC239 is widespread in Europe, Middle East, South and North America, and has been reported from the Asian countries including India [11, 12, 27]. CC239 was assigned to maximum (n = 6) PFGE clusters. All the isolates were frequently resistant to the antibiotics CIP, GEN, AMC, CXM, ERY, CLI, LEV, SXT and only a few ST-239 isolates exhibited resistance to DOX (12.12%), AMK (22.24%). Irrespective of the source of isolation, ST239 strains have been reported to be associated with certain features. These include presence of certain antibiotic resistance genes erm(A), erm(C), aacA-aphD, aadD, aphA3, sat, dfrA, mupA, tet(K), cat, qacA and qacC. The American Academy of Orthopedic Surgeons recommends the use of cefuroxime with vancomycin as antimicrobial prophylaxis for arthroplasty procedures [28] but the present study indicates that MRSA isolates from all the six cases of arthroplasty procedures belong to ST239 and were resistant to cefuroxime.

Also previous reports indicate the prevalence of most common toxin gene combination in ST239 strains with three SE genes *sea*-*sek*-*seq* [12]. Usually, sea-sek-seq is clustered in phage φSa3mu, indicating its possible existence in ST239 strains 20. Indeed PVL and *tsst* was not detected in any of the isolates. Present study indicates that ST239 isolates from West Bengal, India examined are also associated with the same features [13].

The second predominant clonal complex in this study belonged to ST22-MRSA-IV. Unlike ST239 isolates which were highly resistant to the antibiotics CLI, ERY, GEN lower proportion of ST22 isolates resistant to these antibiotics with none of the isolates resistant to DOX and AMK (Fig. 1). Resistance to doxycycline and amikacin appears to be emerging in ST239 and ST772 whereas all other isolates continue to be sensitive with these antibiotics. Although it has been shown in earlier reports [13, 14] that ST22 strains were relatively antibiotic sensitive the isolates examined in the present study were more multidrug resistant compare to earlier reports. The ST22 is also known as epidemic UK-EMRSA-15, Irish AR06, Barnim Epidemic Strain or Spanish PFGE type E13 or Canadian MRSA-8 has been reported from many Asian countries including India [27]. Similar to previous studies presence of egc cluster, *tsst*, PVL has been shown in ST22-MRSA-IV [19]. As the agr locus has the hypervariable genome and was reported to be associated with clonal family [6, 20] in agreement with that, this study also showed that each CC exclusively belongs to one agr group. Study from Mumbai, India indicated that multidrug-susceptible ST22-MRSA-IV and ST772-MRSA-V may be slowly replacing the multidrug-resistant ST239 MRSA-III in hospitals. But in this study majority of the strains still belongs to nosocomial acquired ST 239.

The ST772-MRSA-V, also known as the Bengal Bay clone because of its origin in Bangladesh and India, has been suggested as emerging to become the dominant MRSA clone in India [13, 14]. However, in present study this clone was present in a small minority (4%) of the strains examined.

Interestingly, for the first time in this study ST107 MRSA has been reported which is similar to MRSA C18 strain that was first reported from respiratory isolate in Florida in 1997. Another ST 107 MSSA variant from wound isolates was reported in United States in 2014 [29]. Thus, globally this is the second report of ST107 MRSA clinical isolate. Another known ST642 strain is also been reported first time from India.

## Conclusion

The study could have larger sample size and more generalizability had it been a multicenteric study. However, given that Medical College & Hospital, Kolkata is one of the major tertiary care hospital in Eastern India which serves as a referral centre with high patient load and active post discharge follow up of patients, it is considered that this selection bias is minimal.

Thus, findings of this study establishes MRSA ST239 as a significant pathogen in implant based early POWI and is associated with high rate of virulence, drug resistance, longer duration of hospital stay, implant failure and delayed cure. This study shows that hospital associated MRSA ST239 with new emergence of ST642 and ST107 strains continued to be prevalent in this hospital setup whereas in other parts of India it is being replaced by community associated MRSA strains. Therefore, it is of utmost importance to formulate treatment strategies and antibiotic policy in accordance to epidemiology of hospital microflora especially in case of multidrug resistant MRSA strains.
